# Severe status epilepticus induced by shunt malfunction: A case report

**DOI:** 10.1016/j.heliyon.2025.e42918

**Published:** 2025-02-21

**Authors:** Mohammadreza Moznebiisfahani, Navid Askariardehjani

**Affiliations:** Department of Neurosurgery, School of Medicine, Isfahan University of Medical Sciences, Isfahan, Iran

**Keywords:** Seizure, Shunt malfunction, Foreign body

## Abstract

**Introduction:**

Focal impaired awareness seizures are a common neurological disorder characterized by abnormal electrical activity in a specific brain region, resulting in impaired consciousness and neurological symptoms. Treatment options for patients may include antiepileptic medications, lifestyle modifications, or, in some cases, surgical intervention aimed at resecting the area of the brain responsible for seizure generation. Shunt malfunction can lead to seizures in cases of hydrocephalus; however, seizures triggered by stimulation of a specific brain area by the shunt catheter post-placement have not been reported.

**Case:**

This case provides a compelling example of a seizure triggered by the stimulation of a foreign body in the ventricle. The patient is a 31-year-old man who suffered seizures due to the proximal catheter remaining in the ventricle.

**Conclusion:**

In general, accidental events during surgery should be carefully investigated and taken into consideration. And if necessary, timely intervention should be done to eliminate possible complications.

## 1 Introduction

Focal impaired awareness seizures are a common neurological disorder characterized by abnormal electrical activity in a specific brain region, resulting in impaired consciousness and neurological symptoms [1]. These seizures can manifest in various ways, often leading to alterations in perception, emotion, and behavior. During an episode, individuals may display signs of confusion, unresponsiveness, or involuntary movements [2]. The duration of these seizures typically ranges from a few seconds to several minutes, and they may occur sporadically or in clusters [3]. The underlying causes of focal impaired awareness seizures can vary, including structural brain abnormalities, genetic predispositions, or previous head injuries [4]. Diagnosing these seizures often involves a comprehensive evaluation that includes a detailed medical history, neurological examinations, and diagnostic imaging techniques such as MRI or EEG recordings that capture the brain's electrical activity [5]. Treatment options for patients may include antiepileptic medications, lifestyle modifications, or, in some cases, surgical intervention aimed at resecting the area of the brain responsible for seizure generation [6]. Additionally, ongoing research is focused on better understanding the mechanisms behind focal impaired awareness seizures, with the hope of developing more effective therapies and interventions tailored to individual needs [7]. Shunt malfunction can lead to seizures in cases of hydrocephalus; however, seizures triggered by stimulation of a specific brain area by the shunt catheter post-placement have not been reported. Various studies have reported an increased risk of epileptic seizures after shunt placement, but the underlying mechanisms are still controversial and no proven mechanism has been reported. Direct damage to brain tissue at the time of ventricular catheter placement, the presence of the shunt tube itself as a foreign body, the number of shunt revisions after failure, the occurrence of meningitis and hydrocephalus may be associated with the occurrence of epilepsy [8].

This case is a compelling and rare example of seizures caused by stimulation of a foreign body in the ventricle, which resolved following the removal of the object.

### 1.1 Case

#### 1.1.1 Patient presentation

**Fig. 1:**
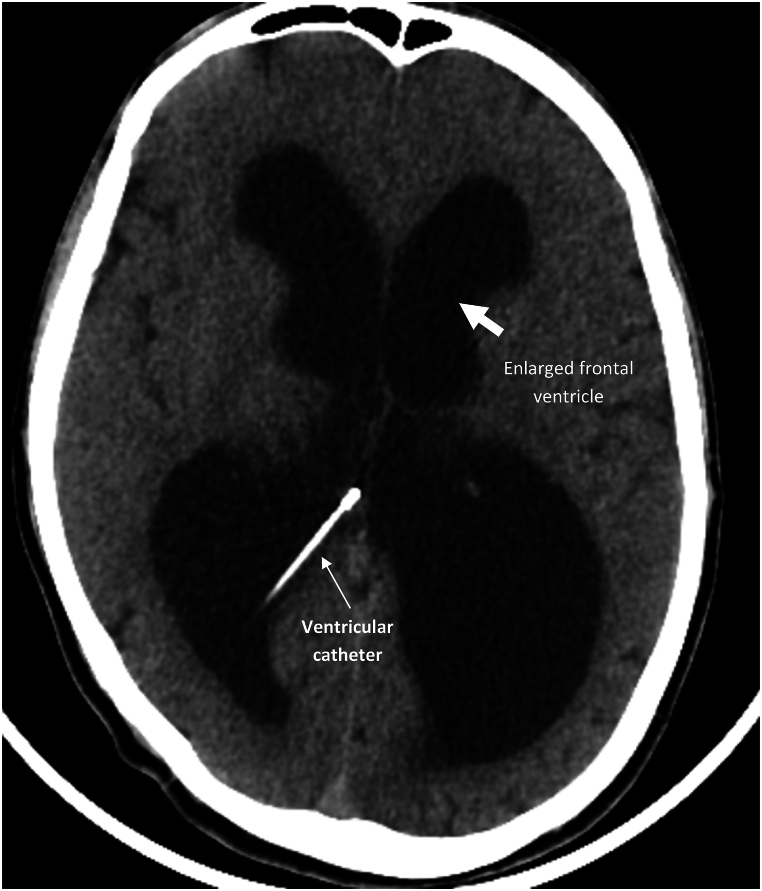
Axial views of CT scan showing the large size of the ventricles and hydrocephalus.

The patient is a 31-year-old man with a history of head trauma six years ago. He was discharged without specific symptoms at the time of the injury. However, a month later, he began experiencing functional difficulties in daily tasks and forgetfulness. The CT scan revealed acute hydrocephalus, leading to a diagnosis of post-traumatic hydrocephalus and subsequent ventriculoperitoneal shunt implantation. After 6 years, the patient presented to the emergency room with loss of consciousness. Initial examination revealed a GCS of 13, and a CT scan showed evidence of hydrocephalus (Fig. 1). Due to the acute loss of consciousness and suspected shunt malfunction, the patient was transferred to the operating room for shunt replacement. During shunt replacement, the proximal shunt catheter was suddenly detached and pulled into the ventricle. And the catheter remained in the ventricle. After the shunt was fully replaced and its functionality verified, the patient was transferred to recovery room.

The day after surgery, the patient was fully awake and exhibited no neurological deficits. However, a few hours later, the patient experienced a unilateral tonic-clonic seizure that progressed to status epilepticus, unresponsive to initial doses of diazepam and phenobarbital. Control was eventually achieved with midazolam, and the maximum permitted doses of levetiracetam, lacosamid, gabapantin and sodium valproate were administered. The listed anticonvulsant medications were prescribed according to the American Epilepsy Society guidelines. Initially, various causes of seizures such as electrolyte disturbances or meningitis were considered as differential diagnoses for the patient. CSF analysis and culture were performed, which were reported to be negative.

#### 1.1.2 Clinical evaluation

**Fig. 2:**
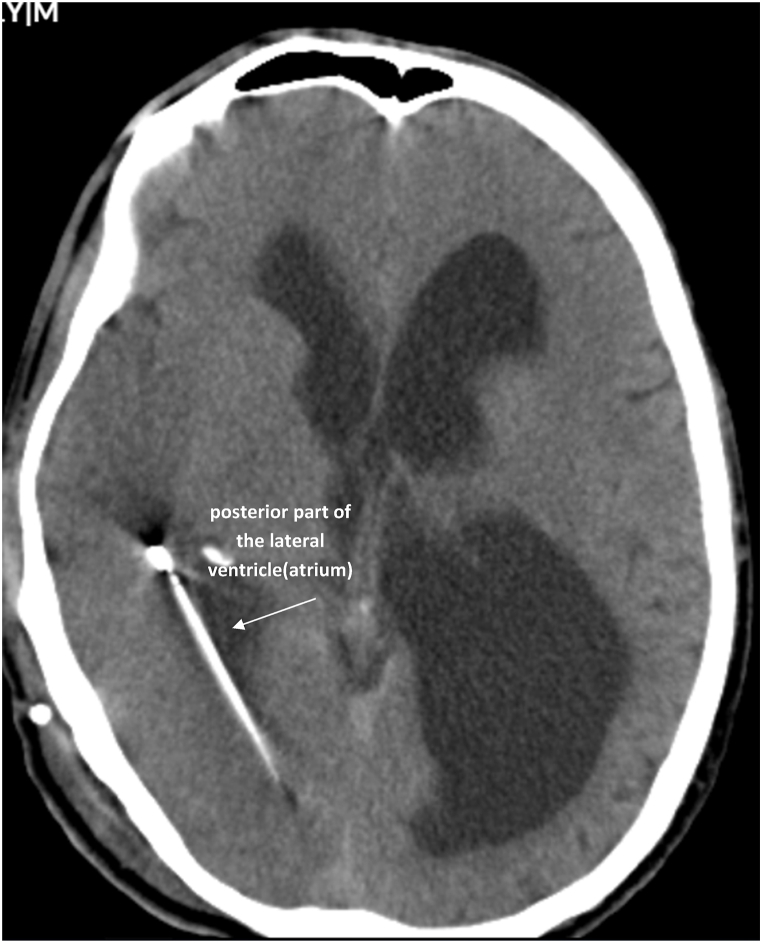
The axial view of the CT scan shows the placement of the ventricular catheter inside the atrium of the right ventricle.

Electrolyte tests for blood sugar, sodium, potassium, calcium, and phosphorus returned normal results. The patient's family had no history of seizures and before the shunt malfunction, the patient had not shown any signs of seizures. An emergency CT scan was ordered for the patient. The CT scan revealed that the shunt catheter had turned from the foramen of Monro towards the temporal horn and was positioned in the atrium of the ventricle, causing pressure on the right temporoparietal lobe of the brain (Fig. 2). Considering that the patient had no previous history of seizures and the patient's clinical presentation was tonic-clonic seizures on the left side, the cause of the seizures was diagnosed as the pressure effect of the shunt catheter on the right temporoparietal lobe.

#### 1.1.3 Surgical intervention

Based on this diagnosis, the patient underwent re-surgery using an endoscope. During the surgery, the CSF sample was sent to the laboratory, which was normal for analysis and culture. During the endoscopy, the catheter was initially visible upon entering the ventricle but disappeared before it could be removed. After searching the frontal ventricle, it was located and successfully extracted. Endoscopy was performed through the previous shunt route. Then the proximal part of the shunt was placed inside the ventricle.

#### 1.1.4 Post operative outcome

The day after surgery, the patient was fully awake. The anticonvulsant drugs were gradually reduced and after six months the patient uses only minimal doses of lacosamide and lotirastam. The patient did not have any seizures during the six-month follow-up. Post-operative imaging confirmed proper placement of the shunt within the ventricle, showing no signs of obstruction or complications. The patient's recovery progressed smoothly, and vital signs remained stable. During the follow-up visits, the neurologist conducted thorough assessments, monitoring for any signs of neurological deficits. The patient reported improvements in overall well-being and cognitive function, with no recurrence of headaches or symptoms experienced prior to the surgery.

The treatment process was clearly explained to the patient, who, along with his family, expressed concern about the shunt catheter remaining in the ventricle. However, after a thorough explanation from the surgeon, they agreed to proceed with the treatment.

Finally, written consent was obtained from the patient for this case report.

## 2 Discussion

According to the studies, any pressure effect on the cortex and deep tissues of the brain can cause neurological symptoms and defects in patients. In most cases, focal seizures are caused by stimulation of a specific part of the brain [9]. As mentioned in this case, the unilateral seizure of the patient was caused by a foreign body inside the ventricle, which caused a compressive effect on the right temporoparietal lobes. According to the studies, any pressure effect on the cortex and deep tissues of the brain can cause neurological symptoms and defects in patients [10]. In most cases, focal seizures are caused by stimulation of a specific part of the brain [11]. Focal impaired awareness seizures are a common neurological disorder characterized by abnormal electrical activity in a specific brain region [12]. As mentioned in this case, the unilateral seizure of the patient was caused by a foreign body inside the ventricle, which caused a compressive effect on the right temporoparietal lobes. This compressive effect not only disrupts normal neuronal function but can also lead to the creation of a seizure focus, where abnormal electrical discharges become self-sustaining. In this scenario, the presence of the foreign body exacerbates the irritability of the surrounding neuronal tissue, further perpetuating the seizure activity. Patients experiencing focal impaired awareness seizures may present with various symptoms, including alterations in consciousness, unusual motor movements, or autonomic changes such as sweating or flushing. The specific manifestations can vary depending on the cortical area involved. For instance, if the seizure focus is localized within the temporal lobe, patients may exhibit olfactory hallucinations or complex visual experiences, whereas seizures originating in the parietal lobe might provoke sensory disturbances [13].

Consistent with the treatment results in this case, in 2023, a report described a case involving a child with seizures caused by a foreign body in the temporal lobe. The surgical removal of the ring-shaped metal object led to a significant improvement in the child's seizures [14].

A 2018 study on the pathophysiology of seizures showed that delaying seizure treatment reduces the effectiveness of first-line treatments [15]. This study confirms that prompt treatment led to a quick response and resolution of the seizures in this patient, highlighting the critical importance of timely diagnosis and intervention. A delay could have resulted in prolonged seizures and negatively impacted the patient's life.

The management of such seizures necessitates a comprehensive approach aimed at alleviating the underlying cause. Surgical intervention may be indicated for the removal of the foreign body, particularly if it is contributing to refractory seizures or other neurological deficits. Post-surgical strategies could involve antiepileptic medications to stabilize neuronal excitability and prevent recurrence of seizure activity. Based on our experience it is not common for foreign objects to remain inside the ventricle during neurosurgery. Reports have shown that the catheter remaining inside the ventricle will be asymptomatic in most cases. But in the case mentioned, the shunt catheter caused an uncontrollable seizure in the patient, who recovered completely after the surgery. Surgeons should be very careful when replacing the shunt and when removing the pump, because in many cases when removing the pump and due to the formation of fibrotic tissue around it, the shunt catheter is separated from the pump and pulled into the ventricle by the pressure of CSF. In many cases, the patient does not show any specific neurological symptoms after the ventricular catheter remains in place. Surgeons should remove the catheter if the patient has symptoms. And as seen in this case, the symptoms improve dramatically.

### 2.1 Limitations

Given that the catheter remaining in the cerebral ventricle was not easily acceptable to the patient and his companions, they initially did not consent to participate in the study, but were eventually convinced by a full explanation.

## 3 Conclusion

In general, accidental events during surgery should be carefully investigated and taken into consideration. And if necessary, timely intervention should be done to eliminate possible complications. This report should be considered by neurosurgeons, both in terms of the necessary precautions during proximal shunt catheter replacement and in terms of rapid diagnosis and treatment following this complication.

## CRediT authorship contribution statement

**Mohammadreza Moznebiisfahani:** Project administration, Data curation, Conceptualization. **Navid Askariardehjani:** Writing – review & editing, Writing – original draft, Visualization, Validation, Supervision, Resources, Project administration, Investigation, Data curation, Conceptualization.

## Informed consent

Informed consent was obtained from the patient involved in this case report. The patient and their family consented to the publication of all images, clinical data, and other information included in this manuscript.

## Ethical approval

This study was conducted in accordance with the Declaration of Helsinki. Ethical approval was obtained from the appropriate institutional review board (IRB).

## Availability of data and materials

The data that support the findings of this study are available but restrictions apply to the availability of these data, which were used under license for the current study, and so are not publicly available. Data are however available from the authors upon reasonable request and with permission of [Navid Askariardehjani].

## Funding statement

The authors received no financial support for the research, authorship, and/or publication of this article.

## Declaration of competing interest

The authors declare that they have no known competing financial interests or personal relationships that could have appeared to influence the work reported in this paper.
